# Supplementary material for: Content-rich biological network constructed by mining PubMed abstracts
Source: BMC Bioinformatics. 2004 Oct 8;5:147. doi: 10.1186/1471-2105-5-147 (PMC528731; doi:10.1186/1471-2105-5-147)
Supplement: Additional File 2 — The original results of the above study (non-essential files are deleted to keep the file size under the limit set by BMC bioinformatics). [file 1471-2105-5-147-S2.bz2 › chilibotAdditionalFile2/dip05/349ID8910586E4497/html/IL13_IL13RA1.html]

 


 **IL13** and **IL13RA1** 
  
Found 9 abstracts in PubMed, retrieved 05.  
 

 What does Google say? 
 PDF only 
| .edu only 

---

**Interactive relationship** (e.g. stimulation, inhibition, etc)

**Inhibitory relationship**- However, treatment of IL 4 deficient mice with IL 13Ra  [ **IL13RA1** ]  during vvGs immunization reduced IL 5, IL 13  [ **IL13** ] , and eotaxin production and pulmonary eosinophilia after RSV challenge.  Ref: 12574374 J Immunol, 2003
**Neutral relationship**

**Non-interactive relationship** (e.g. studied together, co-existance, homology, etc.)

- **Il13ra1**  mRNA was co expressed with I14ra mRNA in mouse myeloid and natural killer cells on which IL 13  [ **IL13** ]  has been known to act.  Ref: 11003391 Immunogenetics, 2000
- We hypothesized that variants in the  **IL13** ,  **IL13RA1** , and IL4RA genes would be associated with an accelerated rate of decline of lung function among smokers.  Ref: 12594065 Am J Respir Cell Mol Biol, 2003
- We determined the allele frequencies of five polymorphisms in the  **IL13** ,  **IL13RA1** , and IL4RA genes in 588 continuing smokers chosen from the NHLBI Lung Health Study for having the fastest n = 282 and slowest n = 306 5 yr rate of decline of lung function mean change in FEV 1 %predicted yr = 4.1 and 1.1, respectively.  Ref: 12594065 Am J Respir Cell Mol Biol, 2003
- Polymorphisms in the  **IL13** ,  **IL13RA1** , and IL4RA genes and rate of decline in lung function in smokers.  Ref: 12594065 Am J Respir Cell Mol Biol, 2003
- These receptors are composed of IL 13  [ **IL13** ]  receptor IL 13R alpha1  [ **IL13RA1** ] , IL 13Ralpha2, and IL 4Ralpha chains.  Ref: 11939409 Oncol Res, 2000
- IL 13  [ **IL13** ]  and the IL 13  [ **IL13** ]  specific receptor chain IL 13R alpha1  [ **IL13RA1** ]  are frequently expressed in cHL derived cell lines and in RS cells from biopsies of cHL tissues.  Ref: 12152987 Leuk Lymphoma, 2002
